# ANCA-associated vasculitis: a phase-oriented therapeutic framework from immunopathology to precision treatment

**DOI:** 10.3389/fmed.2026.1869684

**Published:** 2026-07-07

**Authors:** Elena Treppo, Simone Longhino, Benedetta Fazzi, Laura Di Centa, Cinzia Fabro, Luca Quartuccio

**Affiliations:** Rheumatology Division, Department of Medicine, Academic Hospital “Santa Maria della Misericordia,” Azienda Sanitaria Universitaria Friuli Centrale (ASUFC), University of Udine, Udine, Italy

**Keywords:** ANCA-associated vasculitis, complement inhibition, consolidation, glucocorticoid-sparing agents, maintenance, precision medicine, remission, rituximab

## Abstract

Antineutrophil cytoplasmic antibody (ANCA)-associated vasculitides are rare, severe autoimmune diseases characterized by necrotizing small-vessel inflammation, heterogeneous organ involvement, and a substantial burden of relapse, damage, and treatment-related toxicity. Although their management has traditionally been framed around the dichotomy between remission induction and maintenance, this model does not fully capture the dynamic immunopathological transitions that occur across the disease course. In this review, we propose a phase-oriented therapeutic framework that integrates disease stage, dominant pathogenic mechanisms, organ-specific risk, and unmet clinical needs to support a more precise and clinically actionable approach to treatment. Within this model, the induction phase is focused on rapid control of active inflammation and prevention of irreversible organ damage, with treatment intensity increasingly informed by clinical severity, renal histopathology, complement activation, ANCA specificity, and patient-level vulnerability to toxicity. The consolidation phase represents a biologically active post-induction window in which clinical remission must be stabilized, residual autoreactive immune circuits further suppressed, and glucocorticoids tapered. Rituximab-based strategies exemplify this concept by sustaining B-cell depletion after remission induction rather than replacing the pathogenic target with non-specific immunosuppression. The maintenance phase is then reframed as a period of individualized surveillance, relapse prevention, toxicity minimization, and selective de-escalation. For eosinophilic granulomatosis with polyangiitis, a purely phase-based model is insufficient, and treatment should also account for the relative contribution of eosinophilic inflammation and autoimmune vasculitis, although it should not be reduced to ANCA status alone. Overall, this framework shifts AAV management from a rigid induction–maintenance sequence toward a mechanism-based strategy aimed at matching therapeutic ambition to immunopathology, evidence strength, and patient-specific risk.

## 1 Introduction

Antineutrophil cytoplasmic antibody (ANCA)–associated vasculitides (AAV) comprise a group of rare, potentially life-threatening autoimmune diseases characterized by necrotizing inflammation of small- to medium-sized blood vessels. The main clinical entities include granulomatosis with polyangiitis (GPA), microscopic polyangiitis (MPA), and eosinophilic granulomatosis with polyangiitis (EGPA), which share overlapping pathogenic mechanisms but display distinct clinical and immunological features.

Over the past decades, substantial progress has been made in understanding the immunopathogenesis of AAV. Central to disease development is the loss of immune tolerance leading to the production of ANCAs, primarily directed against proteinase 3 (PR3) and myeloperoxidase (MPO). These autoantibodies play a pathogenic role by activating primed neutrophils, inducing endothelial damage, and promoting the formation of neutrophil extracellular traps (NETs), which further amplify inflammation and autoimmunity. In addition to neutrophil-mediated injury, growing evidence highlights the contribution of the complement system—particularly the alternative pathway and C5a–C5a receptor axis—as well as dysregulated B- and T-cell responses (1).

Recent insights have also emphasized the heterogeneity of AAV, suggesting that PR3-ANCA and MPO-ANCA vasculitis may represent distinct disease subsets with different genetic backgrounds, clinical courses, and relapse risks. Rare mass-forming variants of GPA further illustrate this diagnostic heterogeneity, as they may mimic malignancy or other granulomatous disorders and require biopsy-supported reassessment for correct classification (2). This paradigm shift has important implications for disease classification, prognostication, and therapeutic decision-making.

From a therapeutic perspective, the management of AAV has evolved remarkably. The introduction of cyclophosphamide-based regimens dramatically improved survival, transforming AAV from a fatal condition into a chronic relapsing disease (3). More recently, targeted therapies such as the anti-CD20 monoclonal antibody rituximab have demonstrated non-inferiority—and in some settings superiority—to cyclophosphamide for remission induction, particularly in relapsing disease (4). Furthermore, advances in the understanding of complement activation have led to the development of novel agents such as avacopan, an oral C5a receptor inhibitor, which has shown efficacy in reducing glucocorticoid exposure while maintaining disease control in GPA and MPA (5). On the other hand, the development of drugs targeting interleukin-5 (IL-5) or its receptor has proven particularly effective in EGPA, reflecting the central role of eosinophils in disease pathogenesis. In this setting, agents such as mepolizumab and benralizumab have demonstrated the ability to reduce disease activity, decrease relapse rates, and facilitate glucocorticoid tapering, marking a significant step toward more targeted and less toxic therapeutic strategies (6, 7).

Despite these advances, several unmet needs remain (1). These include the early recognition of subsets of patients with relapsing or refractory disease, the need for reliable biomarkers of disease activity and predictors of relapse, as well as the long-term management of potential adverse effects of therapies, infections, and cardiovascular complications.

Integrated Italian clinical-administrative data have also shown that AAV, despite its rarity, carries substantial hospitalization and healthcare costs, reinforcing early diagnosis and optimized treatment as persistent unmet needs (8).

In this review, we propose a phase-oriented framework integrating disease stage, pathophysiology, and unmet clinical needs, with the aim of providing a more clinically actionable perspective on current and emerging therapies in AAV.

## 2 Rethinking AAV management: a phase-oriented model

Traditional management of AAV has relied on a dichotomous distinction between remission induction and maintenance (9). However, this model does not fully capture the dynamic nature of immune dysregulation and relapse risk in AAV. We propose a three-phase model—induction, consolidation, and maintenance—each characterized by distinct pathophysiological mechanisms, clinical priorities, and unmet needs, which should guide therapeutic choices. The rationale for introducing the concept of a distinct consolidation phase in the therapeutic framework of AAV is grounded in the dissociation between clinical remission and extinction of the pathogenic autoimmune program. In AAV, remission after induction does not necessarily imply eradication of residual autoreactive immunity, as ANCA-producing or ANCA-supporting B-lineage compartments may persist despite apparent disease control. Highly sensitive flow-cytometric studies have shown that rituximab markedly depletes circulating B cells but may not fully eliminate residual B-cell populations, while persistence or re-emergence of autoreactive plasmablasts after B-cell depletion has been associated with subsequent relapse (10, 11). Relapses after rituximab also frequently occur in the setting of B-cell reconstitution, particularly in PR3-ANCA–positive disease. These observations support the view that the early post-induction period is a biologically active window, during which pathogenic immune circuits may be further suppressed or may re-expand, rather than a passive interval of conventional maintenance therapy (12, 13). Accordingly, a phase-oriented framework may provide a more coherent rationale for positioning emerging therapies, linking their mechanisms of action to the specific biological and clinical needs of each disease stage rather than considering them in isolation. Furthermore, in clinical practice, the therapeutic trajectory of AAV begins even before treatment initiation. The transition from suspected disease to treatment allocation is shaped by diagnostic probability, organ-threatening features, histopathology when available particularly when kidney involvement is present, ANCA serotype, complement activation and the competing risks of irreversible damage, infection, and treatment toxicity. Patient profiling is particularly relevant because the same clinical syndrome may reflect different balances between potentially reversible active inflammation and established irreversible damage, which may require different levels of induction intensity and different expectations regarding consolidation or long-term maintenance (14, 15).

## 3 Induction phase: rapid disease control vs. early toxicity

### 3.1 Key unmet needs

- Rapid control of inflammation
- Prevention of irreversible organ damage
- Reduction of glucocorticoid toxicity
- Identification of patients needing intensified therapy

### 3.2 Current strategies in GPA and MPA

The goal of remission induction in AAV is the rapid control of inflammation in order to prevent irreversible organ damage and reduce early mortality. Therapeutic strategies are primarily guided by disease severity, organ involvement, ANCA specificity, and patient-related factors, including age, comorbidities, frailty, and infectious risk. In this context, particularly when renal involvement is present, histopathology plays a crucial role, and kidney biopsy should be regarded not only as a diagnostic procedure but also as a tool for therapeutic profiling. The Berden histopathological classification stratifies ANCA-associated glomerulonephritis into focal, crescentic, mixed, and sclerotic classes according to the predominant pattern of glomerular injury, with these categories being associated with distinct renal outcomes (14). The Renal Risk Score further refines prognostic stratification by integrating the percentage of normal glomeruli, the extent of tubular atrophy/interstitial fibrosis, and baseline estimated glomerular filtration rate to estimate the risk of end-stage kidney disease (14, 15). Accordingly, biopsy findings may help identify patients with predominantly active and potentially reversible lesions. This is particularly relevant when crescentic lesions are prominent or when adverse prognostic features, such as fibrinoid necrosis, are present. In these patients, intensified induction may be justified. Conversely, extensive chronic scarring, such as > 33% globally sclerotic glomeruli or significant interstitial fibrosis, may indicate limited potential for renal recovery. In this setting, treatment escalation is less likely to restore kidney function and may mainly increase treatment-related toxicity (15, 16).

Complement status may further refine this early risk assessment. Although AAV is classically described as a pauci-immune vasculitis, low serum C3 at diagnosis has repeatedly been associated with more severe renal disease, poorer renal survival, and worse patient outcomes (16, 17). In renal AAV, hypocomplementemia may therefore identify a subgroup with stronger complement-driven inflammatory amplification, supporting closer monitoring and, in selected severe renal presentations, a lower threshold for aggressive induction and early consideration of complement-targeted, glucocorticoid-sparing strategies (5, 9, 17, 18).

For patients with severe, organ- or life-threatening GPA/MPA induction generally combines glucocorticoids, preferably using reduced-exposure protocols whenever clinically appropriate, with either rituximab or cyclophosphamide (19). As glucocorticoids remain a cornerstone of induction therapy, current induction DMARDs are outlined in Table 1, highlighting the patient subsets most likely to benefit, along with their potential advantages and limitations.

**TABLE 1:** Current induction DMARDs in AAV: indications, benefits, and limitations.

Therapy	Patient subsets	Pros	Cons
Cyclophosphamide	- Newly diagnosed severe disease - Major organ involvement (kidney, lung)	- High efficacy in remission induction - Strong evidence from landmark trials - Rapid disease control	- Cumulative toxicity - Infertility - Increased risk of infections - Increased risk of malignancy
Rituximab	- Relapsing disease - PR3-ANCA positive patients - Younger patients - Patients wishing to preserve fertility - Prior exposure to cyclophosphamide	- Non-inferior to cyclophosphamide for induction - Superior in relapsing disease - Steroid- and cyclophosphamide-sparing - Better fertility profile	- Risk of infections (including hypogammaglobulinemia) - Need for repeated treatments - Long-term data still evolving

In aggressive and potentially reversible disease, combination therapy with cyclophosphamide and rituximab should be considered, particularly due to the slower onset of action of rituximab. This approach may be especially relevant in patients with severe, organ- or life-threatening manifestations, including rapidly progressive glomerulonephritis, alveolar hemorrhage, mononeuritis multiplex or progressive peripheral neuropathy, myocarditis, gastrointestinal involvement, and central nervous system manifestations, including retinal vasculitis.

Glucocorticoids remain a key component of induction regimens; however, recent studies such as the LoVAS and PEXIVAS trials have supported reduced-dose steroid protocols to minimize toxicity without compromising efficacy (19, 20). In PEXIVAS, which enrolled patients with severe AAV, defined by markedly impaired renal function or diffuse pulmonary hemorrhage, a reduced-dose oral glucocorticoid regimen was non-inferior to a standard-dose regimen for the composite outcome of death or end-stage kidney disease, while being associated with a lower rate of serious infections during the first year (19). This provided robust evidence that steroid exposure can be substantially reduced even in severe disease without compromising major hard outcomes. The LoVAS trial extended this concept to a rituximab-based induction strategy in newly diagnosed AAV without severe glomerulonephritis or alveolar hemorrhage: reduced-dose prednisolone (0.5 mg/kg/day) plus rituximab was non-inferior to conventional high-dose prednisolone plus rituximab for remission induction at 6 months, with fewer serious adverse events and serious infections (20). Importantly, the predefined 24-month follow-up showed comparable relapse frequencies between reduced- and high-dose glucocorticoid regimens, while serious adverse events remained less frequent in the reduced-dose arm (21). Taken together, PEXIVAS and LoVAS support a move from uniform high-dose glucocorticoid exposure toward individualized steroid minimization, calibrated according to disease severity, organ-threatening manifestations, concomitant use of rituximab, and patient-level vulnerability to treatment-related toxicity.

In this context, the use of avacopan, an oral C5a receptor inhibitor, represents a major advancement with a mechanistically distinct approach. By targeting the C5a-C5aR1 axis, avacopan attenuates complement-driven neutrophil activation and allows for significant glucocorticoid sparing while maintaining disease control, as demonstrated in the ADVOCATE trial (5). Avacopan is currently approved for use only during the remission induction phase combined with rituximab or cyclophosphamide and for a duration of up to 1 year, as the optimal duration beyond the first year, the consequences of discontinuation, and the best strategy for re-treatment after relapse have not been defined (5). Longer-term safety also requires continued pharmacovigilance, particularly for hepatic abnormalities, serious infections, relapse after withdrawal, and use beyond 12 months in real-world practice (22–25). Specifically, hepatic safety of avacopan is the most important evolving signal. In the ADVOCATE registration dataset and pooled pre-registration safety analyses, liver enzyme elevations were overall similar between avacopan- and prednisone-based regimens, with ALT/AST increases reported in approximately 12% versus 13% of patients, respectively. However, serious hepatic adverse events with aminotransferases > 3 times the upper limit of normal were numerically more frequent with avacopan than with prednisone-based treatment, occurring in 5.4% versus approximately 3.6–3.7% of patients; in clinical trials, these abnormalities generally resolved after treatment discontinuation (5, 26). Post-marketing and real-world Japanese data appear less reassuring and suggest a substantially higher hepatic susceptibility. In one Japanese cohort, aminotransferase elevations were reported in 6/36 patients (16.7%), while a multicenter Japanese study described drug-induced liver injury in 9/22 patients (40.9%), including severe cholestatic forms and one fatal case of vanishing bile duct syndrome (27, 28). This signal is consistent with the FDA pharmacovigilance communication, which identified 76 post-marketing cases of avacopan-associated DILI, including 74 serious outcomes, 54 hospitalizations, 8 deaths, and 7 biopsy-confirmed cases of vanishing bile duct syndrome, with 66/76 cases reported from Japan (29). Conversely, this magnitude of hepatotoxicity has not been consistently reproduced in Western real-world cohorts. In a US multicenter cohort, avacopan was discontinued because of elevated aminotransferases in 4/92 patients (4.3%), while the Spanish early-access experience reported only two avacopan-related adverse events and no prominent hepatotoxicity signal (23). This geographic clustering suggests a possible host susceptibility, potentially including genetic or pharmacogenetic predisposition. This interpretation is biologically plausible by analogy with idiosyncratic hepatotoxicity described for other immunosuppressive agents, including methotrexate and leflunomide, for which genetic variants have been associated with toxicity risk or treatment discontinuation (30–32). Pending better risk stratification, avacopan use should therefore include baseline liver assessment, close early serial monitoring of liver enzymes and cholestatic parameters, prompt discontinuation in case of significant elevation or cholestatic symptoms, and cautious patient selection, particularly in elderly, low-BMI and Japanese patients. Concerning the long-term use, the AVAC-EUR study is an ongoing European multicenter observational cohort designed to evaluate the real-world use of avacopan in ANCA-associated vasculitis, including treatment beyond 12 months. Preliminary data suggest that prolonged therapy may be associated with lower hospitalization and infection rates (33); however, these findings are based on small, non-randomized cohorts and require confirmation in larger studies. Finally, cost and reimbursement remain important barriers to implementation, since pharmacoeconomic analyses indicate that the value of avacopan is highly dependent on drug price, willingness-to-pay thresholds, and health-system assumptions (34). Current evidence suggests that the patients who may derive the greatest benefit from avacopan are those with more severe disease (e.g., patients with renal involvement) and a high risk of glucocorticoid-related toxicity, rather than a single clearly defined biological subset (35). These data, however, should be interpreted in light of recent regulatory concerns. In addition to emerging post-marketing safety issues, the Center for Drug Evaluation and Research has proposed the withdrawal of avacopan from the U.S. market after concluding that new information raised concerns about the reliability of the pivotal ADVOCATE efficacy dataset (36). Pending completion of this regulatory review, avacopan-related efficacy and safety data should therefore be considered within an evolving benefit–risk framework.

Adjunctive therapies may be considered in selected cases. Plasma exchange is now reserved for carefully selected patients with severe, life- or organ-threatening manifestations of AAV. Following the PEXIVAS trial, its role has been substantially narrowed, and the most recent EULAR recommendations suggest considering plasma exchange only in patients with the concomitant presence of serum anti-glomerular basal membrane (anti-GBM) or with severe renal involvement, such as rapidly progressive glomerulonephritis with markedly reduced kidney function or dialysis dependency (9). This selective use is supported by evidence suggesting a possible reduction in the 12-month risk of end-stage kidney disease, consistent with the ability of plasma exchange to rapidly remove circulating pathogenic antibodies. However, no clear mortality benefit has been demonstrated, and plasma exchange is associated with an increased risk of serious infections during the first year of disease (19, 37). The routine use of plasma exchange for alveolar hemorrhage in GPA and MPA is not currently recommended by EULAR, reflecting the lack of convincing evidence for a clear outcome benefit in this setting (9). Accordingly, plasma exchange should be framed as an individualized adjunctive or rescue strategy rather than a universal component of induction, particularly in patients with dialysis dependency, rapidly progressive renal failure, or the highest short-term risk of irreversible kidney loss (9, 19, 37).

At this stage, the main unmet need is not only how to induce remission, but how to identify the most appropriate treatment intensity for each patient. Nowadays, induction therapy should increasingly move from a severity-only approach toward an integrated clinicopathological and biomarker-driven model. In this model, rapidly progressive renal dysfunction, active biopsy lesions, low complement levels, pulmonary hemorrhage, frailty, infectious risk, and ANCA specificity are considered together to calibrate the balance between undertreatment, irreversible organ damage, and early treatment-related toxicity (14–17, 38). This risk-adapted perspective helps position cyclophosphamide, rituximab, avacopan, and plasma exchange not as competing options in the abstract, but as tools to be selected according to the dominant clinical threat.

### 3.3 Emerging approaches: complement inhibition

Emerging approaches in this phase primarily focus on complement inhibition, targeting the C5a–neutrophil amplification loop. In AAV, complement activation is predominantly driven by the alternative pathway, with C5a playing a central role in amplifying inflammation through neutrophil priming and activation (18).

Targeting the C5a–C5aR1 axis has therefore emerged as a key therapeutic strategy (39). The therapeutic rationale for inhibiting C5a-C5aR1 should not be interpreted as strictly dependent on detectable serum ANCA, because complement activation can function as a downstream inflammatory amplifier of neutrophil activation and vascular injury. However, the pivotal evidence base for avacopan is essentially restricted to ANCA-positive GPA/MPA, and robust clinical data supporting routine use in seronegative AAV are lacking. In ANCA-negative patients with severe vasculitic manifestations, a phase-oriented model should therefore rely more heavily on clinical phenotype, organ-threatening involvement, biopsy findings, complement consumption or other markers of complement activation, and competing toxicity risks rather than ANCA status alone (39). Alternative approaches include vilobelimab, which neutralizes circulating C5a and has shown preliminary encouraging signals in phase II trials (e.g., NCT03895801), supporting the hypothesis of possible use in glucocorticoid-free regimens (40). Compared with receptor blockade, ligand neutralization may allow broader inhibition of C5a-driven inflammation, although this strategy currently lacks phase III validation.

More recently, upstream inhibition of the alternative pathway has been explored with iptacopan, an oral factor B inhibitor that prevents C3 convertase formation and reduces both C3a and C5a generation. This approach may provide a more comprehensive suppression of complement-mediated inflammation, potentially translating into improved disease control, particularly in severe or refractory cases. However, in contrast to the more selective blockade of the C5a–C5aR1 axis, factor B inhibition may also interfere with physiological complement functions, including opsonization and microbial clearance, raising theoretical concerns regarding an increased risk of infections (e.g., need to targeted vaccination against encapsulated bacteria). Clinical evidence in AAV is currently limited, and ongoing studies (e.g., NCT06388941) are evaluating its efficacy and safety.

Conceptually, the induction phase is progressively shifting from broad immunosuppression toward selective targeting of key inflammatory amplification pathways, aiming to achieve rapid disease control while minimizing early treatment-related toxicity. However, outside avacopan, most complement-directed approaches in AAV remain supported by early-phase or ongoing studies, and their clinical positioning should therefore be considered provisional.

## 4 Consolidation phase: stabilizing immune remission

### 4.1 Key unmet needs

- Incomplete B-cell depletion
- Early relapse risk
- Persistence of autoreactive clones
- Steroid tapering

### 4.2 Current strategies in GPA and MPA

The consolidation phase may be defined as the early post-induction interval in which the therapeutic objective is to stabilize a recently achieved remission, prevent rapid re-expansion of autoreactive B-cell clones, and enable glucocorticoid withdrawal before long-term de-escalation becomes the dominant clinical issue. This concept has clear precedents in other fields of medicine: in hematology and oncology, consolidation denotes post-remission treatment delivered after induction to reduce residual disease and relapse risk, with measurable residual disease illustrating that apparent clinical remission may coexist with persistent pathogenic clones (41, 42). Similarly, in transplantation, the distinction between induction immunosuppression, aimed at controlling the early high-risk immune phase, and maintenance immunosuppression, aimed at preventing long-term immune reactivation and rejection, provides a useful conceptual parallel (43, 44). In AAV, this analogy is particularly relevant because clinical remission does not necessarily imply complete immunological remission, and the early post-induction period may still be characterized by residual or reconstituting autoreactive B-cell compartments capable of driving relapse (22). Within this framework, rituximab represents the prototypical consolidation agent, as its use after remission induction directly continues the pathogenic target addressed during the initial phase rather than replacing it with broader, less mechanism-specific immunosuppression. In older treatment paradigms, patients typically transitioned from cyclophosphamide induction to azathioprine maintenance, whereas rituximab-induced remission followed by azathioprine similarly interrupts active targeting of the B-cell compartment (4, 45). By contrast, scheduled post-induction rituximab sustains anti-CD20–mediated B-cell depletion, aiming to convert clinical remission into a more durable immunological remission while glucocorticoids are progressively tapered (46–49). This makes the rituximab-to-rituximab approach a more explicitly pathogenesis-driven strategy, because the mechanism initiated during induction is deliberately consolidated rather than abandoned at the point of clinical response (46–53).

The clinical relevance of this model is supported by randomized trials and long-term cohort data showing that scheduled rituximab after remission induction is more effective than azathioprine in preventing relapse, particularly in relapsing disease and in patients with PR3-ANCA positivity or other relapse-prone features (46–51). Prolonged rituximab treatment may further reduce relapse risk, although this benefit must be balanced against hypogammaglobulinemia, infections, and cumulative immunosuppressive burden (54). Multicenter data indicate that rituximab-associated hypogammaglobulinemia is clinically relevant after rituximab-based induction, while infection-prediction models emphasize that age, renal impairment, glucocorticoid exposure, and previous treatment history should inform decisions on prolonged maintenance. Thus, prolonged B-cell depletion should not be regarded as an indefinitely benign maintenance strategy; it requires baseline and serial immunoglobulin assessment, vaccination planning, infection-risk stratification, and periodic reassessment of whether relapse prevention continues to outweigh treatment-related harm (54, 55).

From a practical perspective, fixed-schedule rituximab is commonly administered every 6 months for at least 18 months after induction, with extension up to 36 months or longer considered in patients at high risk of relapse (46–49). Recent randomized evidence showing fewer relapses when retreatment is guided by B-cell repopulation rather than by ANCA rise further reinforces the biological coherence of this strategy, indicating that the clinically relevant event is not merely serological fluctuation, but the re-emergence of the target B-cell compartment itself (22). Thus, rituximab-based consolidation may be viewed as a time-limited, mechanism-based strategy designed to deepen and stabilize newly achieved remission before transition to individualized long-term maintenance or treatment de-escalation.

### 4.3 Emerging approaches beyond rituximab: next-generation B cell–targeted therapies

Emerging strategies in the consolidation phase are primarily driven by the need to overcome incomplete B-cell depletion, prevent the early reconstitution of autoreactive clones, and suppress persistent autoantibody production. Next-generation B-cell- and plasma-cell–directed therapies are particularly well aligned with this consolidation concept, as incomplete B-cell depletion, especially within tissue compartments, remains a major limitation of current anti-CD20 approaches and may contribute to early relapse. Obinutuzumab, a type II glycoengineered anti-CD20 monoclonal antibody, has been designed to achieve more potent and sustained B-cell depletion through enhanced antibody-dependent cellular cytotoxicity and reduced CD20 internalization. This approach is currently being evaluated in randomized clinical trials, including the phase II ObiVAS study (obinutuzumab vs. rituximab), to test whether deeper B-cell depletion can improve tissue targeting and reduce relapse risk (56). Moreover, unlike rituximab, it acts independently of complement activation, which may already be altered or dysregulated in the disease. While deeper depletion may translate into improved disease control—particularly in relapsing or PR3-ANCA-positive patients—its clinical benefit remains to be established, and increased immunosuppression may be associated with a higher risk of infections and hypogammaglobulinemia.

Following B-cell depletion, increased BAFF levels may promote the re-emergence of autoreactive clones, representing a possible major driver of relapse. This provides the rationale for sequential strategies combining B-cell depletion with BAFF inhibition. The COMBIVAS trial (NCT03967925) evaluates rituximab followed by belimumab, aiming to enhance the durability of remission by limiting pathogenic B-cell reconstitution. However, belimumab alone has shown limited efficacy in AAV, and its benefit appears to depend on combination or sequential use. In addition, delayed onset of action and the risk of additive immunosuppression remain potential limitations.

Persistent autoantibody production may be sustained by plasmablasts and long-lived plasma cells, which are not affected by anti-CD20 therapies. This has led to the development of strategies targeting downstream compartments of the B-cell lineage. Telitacicept, a dual BAFF/APRIL inhibitor, extends its effect to plasmablasts and plasma cells, potentially providing a more comprehensive suppression of autoantibody production. This mechanism may potentially reduce relapse rates more effectively than BAFF inhibition alone, however it remains unproven in AAV. In parallel, case reports have described the use of daratumumab in severe, refractory, and heavily pre-treated AAV, supporting the concept of a plasma cell–driven disease phenotype (57, 58), although no clinical trials are currently ongoing.

Overall, the consolidation phase represents a critical immunological window in which the goal extends beyond maintaining remission to actively reshaping the autoreactive immune repertoire. From a phase-oriented perspective, consolidation is also the stage in which the clinician begins to balance two opposite risks: under-treatment, leading to early relapse, and over-treatment, leading to hypogammaglobulinemia, infections, and cumulative immunosuppressive burden. This trade-off is especially relevant in patients with PR3-ANCA positivity, previous relapsing disease, structural lung damage, or prior infectious complications, in whom deeper or prolonged B-cell suppression may be both more beneficial and more hazardous.

## 5 Maintenance phase: long-term control vs. overtreatment

### 5.1 Key unmet needs

- Relapse prediction
- Treatment discontinuation
- Cumulative toxicity
- Biomarkers

### 5.2 Current strategies in GPA and MPA

In maintenance, the key clinical challenge extends beyond relapse prevention alone. The physician must simultaneously address residual disease activity, chronic glucocorticoid exposure, infectious vulnerability, progressive organ damage, and the uncertainty surrounding treatment discontinuation. Thus, maintenance should be conceptualized not merely as a low-intensity continuation phase, but as a dynamic period of surveillance, de-escalation, and selective re-intervention.

In this context, treatment is guided by biomarkers and clinical monitoring, with the aim of minimizing overtreatment while preventing relapse.

Traditionally, both ANCA titers and peripheral B-cell (CD19^+^) repopulation have been considered as potential triggers for retreatment; however, their relative utility has been debated. More recently, a randomized study by Zonozi et al. demonstrated that a strategy based on B-cell repopulation was associated with fewer relapses compared to retreatment guided by ANCA rise, supporting the concept that CD19^+^ B-cell monitoring may be a more reliable biomarker for guiding therapy (22). These findings reinforce the idea that maintaining B-cell depletion—rather than reacting to serological flares—may better control disease activity, although optimal thresholds and timing for retreatment remain to be fully defined.

Although no clearly defined subset of patients with AAV has been identified in whom immunosuppressive therapy can be safely discontinued, relapse risk may persist even after prolonged periods of clinical remission. ANCA specificity remains one of the most informative predictors of disease course, with PR3-ANCA positivity consistently associated with a higher relapse risk than MPO-ANCA positivity, whereas severe renal involvement is generally associated with a lower tendency to relapse but a greater risk of irreversible damage when relapse occurs (38, 59). In MPO-ANCA-associated glomerulonephritis, patients who become and remain MPO-ANCA negative after remission induction appear to have a particularly low risk of relapse, even in the absence of conventional maintenance therapy, while persistent or recurrent MPO-ANCA positivity identifies a higher-risk subgroup (59). Recent *post-hoc* analyses from the RITAZAREM trial further suggest that predictors of relapse may vary according to treatment phase. During maintenance therapy, musculoskeletal involvement and higher patient global assessment scores were associated with subsequent relapse, whereas following treatment withdrawal, B-cell repopulation and ANCA reappearance emerged as the strongest predictors of disease recurrence. These findings support a more individualized approach to treatment de-escalation and suggest that monitoring immune reconstitution biomarkers may help identify patients at increased risk of relapse after discontinuation of maintenance therapy (60). Consequently, prolonged B-cell-depleting strategies may be most justified in PR3-ANCA-positive, relapsing, persistently ANCA-positive, or B-cell-repopulating patients, whereas carefully selected MPO-ANCA-positive patients with renal-limited disease, sustained serological remission, stable kidney function, and no relapse-prone extrarenal phenotype may be candidates for a more restrained de-escalation strategy (13, 38, 59).

### 5.3 Novel approaches: from chronic immunosuppression toward precision immunomodulation and immune system reset

The identification of reliable biomarkers to guide treatment decisions represents a major unmet need in AAV. Reliable biomarkers may reduce overtreatment while maintaining disease control.

An alternative strategy may be to directly reduce circulating pathogenic autoantibodies, potentially reducing the burden of long-term immunosuppression. In this context, FcRn inhibitors such as efgartigimod accelerate IgG degradation by blocking neonatal Fc receptor–mediated recycling, leading to a rapid decrease in ANCA levels. While clinical evidence in AAV is currently limited, a recent case report described its use in a patient with severe, refractory p-ANCA–associated vasculitis with prominent cutaneous involvement (61). In this setting, efgartigimod was administered off-label following failure of standard therapies, including high-dose glucocorticoids, cyclophosphamide, and plasma exchange, leading to a rapid and marked improvement in skin manifestations without significant adverse events. As these findings are preliminary and derived from a single case, they should currently be interpreted as biological proof of concept rather than evidence supporting an established therapeutic option. They nevertheless support the rationale for targeting IgG recycling pathways in AAV and suggest that FcRn antagonists may, in the future, represent adjunctive or rescue therapies in selected patients with refractory disease. Controlled clinical trials are required to define their efficacy, safety, and optimal positioning within the therapeutic landscape of AAV. Key unanswered questions include the magnitude and durability of ANCA reduction, the need for combination with background immunosuppression, the risk of relapse after IgG recovery, infection susceptibility, and the clinical phenotype in which transient IgG lowering may be preferable to deeper B-cell or plasma-cell depletion.

In addition, emerging cellular therapies such as CAR-T cells introduce the concept of immune reprogramming. By targeting CD19, CAR-T cells induce deep depletion of the B-cell lineage, including plasmablasts, leading not only to a marked reduction in autoantibody production but also to the reconstitution of a naïve, non-autoreactive B-cell repertoire. In patients with relapsing or refractory AAV, CAR-T cell therapy represents a promising but still investigational strategy. Emerging evidence supports its biological rationale: in a murine MPO-AAV model, CD19-directed CAR-T cells prevented glomerulonephritis and reduced MPO-ANCA levels, while three published cases of refractory AAV reported rapid clinical remission, BVAS reduction to 0, marked ANCA reduction or clearance, and sustained B-cell aplasia with an overall manageable short-term safety profile (62, 63). However, no controlled trials have yet defined its efficacy, durability, retreatment strategies, or comparative safety versus repeated rituximab exposure. Key unresolved issues include cytokine-release syndrome, immune-effector-cell-associated neurotoxicity, prolonged B-cell aplasia, hypogammaglobulinemia, infections, and major cost and feasibility constraints.. In particular, individualized leukapheresis and manufacturing, bridging therapy, lymphodepletion, inpatient monitoring for cytokine-release syndrome/neurotoxicity, availability of accredited cell-therapy units, trained multidisciplinary teams, prolonged infection surveillance, and costs far exceeding rituximab or other biologic strategies may limit its applicability in the short term. These aspects need to be carefully addressed before CAR-T cell therapy can be positioned within the therapeutic landscape of AAV, because since AAV already has effective non-cellular options, the threshold for CAR-T use will likely remain restricted to truly refractory, organ-threatening disease until controlled durability, safety and pharmacoeconomic data are available (63). Several early-phase clinical trials are currently ongoing to clarify these questions (e.g., NCT06590545, NCT06868290, NCT06685042, and NCT06375993).

Together, these strategies highlight a paradigm shift in the maintenance phase—from chronic immunosuppression toward precision immunomodulation and, potentially, immune system reset.

**FIGURE 1:**
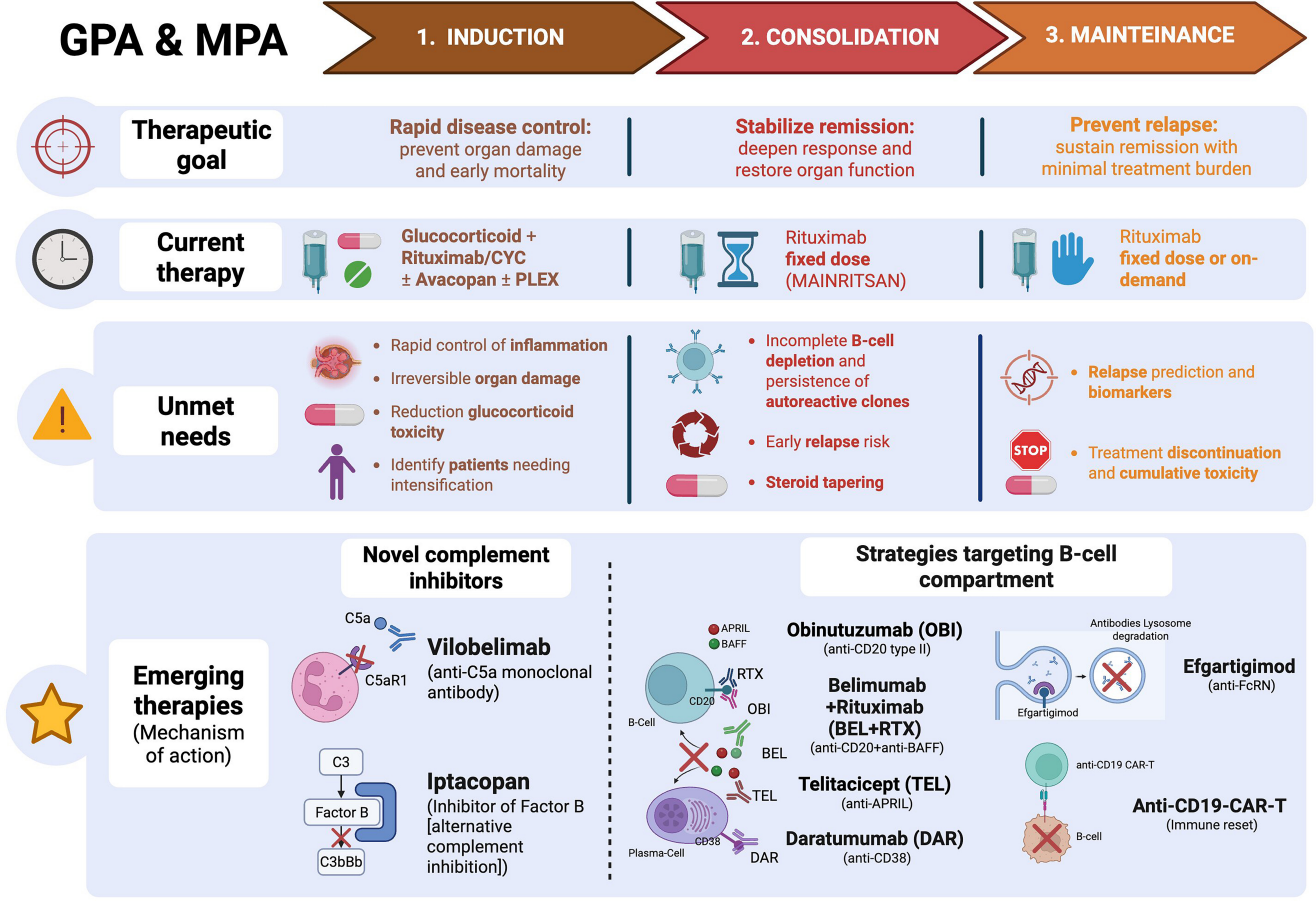
Phase-oriented therapeutic framework for granulomatosis with polyangiitis (GPA) and microscopic polyangiitis (MPA). CYC, Cyclophosphamide; PLEX, plasma exchange.

Figure 1 summarizes a phase-oriented therapeutic framework for GPA and MPA, integrating the therapeutic goals, current standard of care, major unmet needs, and the main emerging treatment strategies across the induction, consolidation, and maintenance phases.

## 6 EGPA: a phase- and phenotype-integrated model

EGPA differs from other AAV in that its pathogenesis is driven by the coexistence of two partially overlapping but distinct mechanisms: eosinophil-mediated inflammation and autoimmune vasculitis. As a result, a purely phase-based therapeutic model may be insufficient to capture the complexity of the disease. This dual-pathway model provides a rationale for a phase- and phenotype-integrated approach, in which treatment is tailored not only to disease activity (induction, consolidation, maintenance) but also to the predominant immunological driver.

### 6.1 Key unmet needs

- Phenotype stratification
- Incomplete response to IL-5–targeted therapies
- Glucocorticoid dependence
- Limited data on combination therapies

### 6.2 Current strategies in EGPA

In EGPA, the induction phase is primarily aimed at achieving rapid disease control, typically through high-dose glucocorticoids, with the addition of immunosuppressive agents such as cyclophosphamide in patients with severe or organ-threatening manifestations (9, 64, 65). Rituximab should be positioned more cautiously and more mechanistically in this setting: it may be considered in selected patients with a predominantly vasculitic phenotype, particularly with MPO-ANCA positivity, although vasculitic EGPA is not synonymous with ANCA positivity. Definite histopathological vasculitic features can occur in ANCA-negative patients, including cardiac and pulmonary presentations; therefore, treatment allocation should integrate organ involvement, biopsy/imaging evidence, Five-Factor Score, eosinophilic burden and ANCA status rather than ANCA status alone (66–68). Moreover, in the REOVAS trial, rituximab was not superior to conventional FFS-stratified therapy for remission induction. In the FFS ≥ 1 subgroup, available data do not demonstrate a clear advantage of rituximab over cyclophosphamide-based standard therapy, although MPO-ANCA-positive patients may experience fewer relapses with rituximab during follow-up in retrospective studies (65, 69); this remains hypothesis-generating rather than practice-defining.

Once remission is achieved, the EGPA consolidation phase should be defined as a biologically active steroid-withdrawal window rather than as maintenance in miniature. Unlike GPA/MPA, where consolidation is mainly linked to suppression of residual autoreactive B-cell activity, EGPA consolidation is anchored in maintaining control of eosinophilic/type 2 inflammation and, in selected cases, residual vasculitic activity while glucocorticoids are tapered. In this phase, the introduction of targeted therapies against IL5-IL5R axis such as mepolizumab (anti-IL5) or benralizumab (anti IL5R) plays a key role, as because it dictates the survival, maturation, and activation of eosinophils, allowing for effective disease control while facilitating steroid tapering. Mepolizumab and benralizumab are central options for relapsing/refractory or glucocorticoid-dependent EGPA, particularly when asthma, ENT disease, eosinophilia or other Th2 manifestations dominate; mepolizumab reduced relapses and enabled steroid reduction in MIRRA trial, while benralizumab was non-inferior to mepolizumab in MANDARA with near-complete eosinophil depletion and greater glucocorticoid withdrawal than mepolizumab (6, 7). Emerging data suggest that a combined or sequential approach targeting both B cells and eosinophils may be beneficial in highly selected patients with refractory or relapsing disease and mixed phenotypes. Markers of eosinophil activation may help refine the consolidation phase biologically, but they remain exploratory. Serum eosinophil cationic protein (ECP) reflects eosinophil degranulation and has been reported to correlate with disease activity and blood eosinophil counts in EGPA; IL-5 and urinary eosinophil-derived neurotoxin have also been proposed as activity markers (70, 71). Nevertheless, ECP is not standardized for routine decision-making, may be influenced by uncontrolled asthma/atopy, sampling and assay variability, glucocorticoids and anti-IL-5/IL-5R treatment, and has not been validated to guide biologic selection, combination therapy, tapering or withdrawal. More broadly, longitudinal biomarker studies in established EGPA have shown that currently available serum biomarkers do not reliably discriminate active from inactive disease during glucocorticoid exposure (72).

Finally, the maintenance phase focuses on sustaining remission with the minimal effective therapy, often continuing biologic agents in responders and adopting a personalized, phenotype-driven strategy based on eosinophilic versus vasculitic features. Maintenance should therefore be phenotype-driven but repeatedly re-anchored to objective manifestations: anti-IL-5/IL-5R therapy may leave vasculitic disease undertreated, whereas rituximab may leave eosinophilic airway disease active; systematic reassessment is more appropriate than a fixed ANCA-based algorithm.

### 6.3 Emerging approaches in EGPA

Beyond established IL-5–targeting agents, several novel or repurposed biologic strategies are under investigation in EGPA, mainly aiming to improve steroid sparing, control refractory airway/ENT disease, and address heterogeneous type 2 inflammatory pathways. Depemokimab is a next-generation anti–IL-5 monoclonal antibody with prolonged IL-5 binding and an extended dosing interval. Its potential advantage is therefore not a different biological target, but a more convenient and sustained suppression of IL-5–driven eosinophilic inflammation. A model-informed development strategy selected depemokimab 200 mg subcutaneously every 26 weeks for EGPA, and the phase III OCEAN trial is currently comparing depemokimab with mepolizumab 300 mg every 4 weeks in adults with relapsing or refractory EGPA receiving standard of care (73) (NCT05263934). Reslizumab, administered intravenously, has shown efficacy in eosinophilic asthma and has been explored in small studies and early-phase trials in EGPA (e.g., NCT02947945) (74). Although reslizumab shares a similar mechanism of action with mepolizumab, its intravenous administration enables weight-based dosing (3 mg/kg), may result in greater systemic bioavailability, and allows for hospital-based delivery with closer clinical monitoring, which can be beneficial in complex or poorly adherent patients. Whether these pharmacological and logistical advantages translate into superior outcomes in EGPA remains uncertain and requires confirmation in adequately powered studies.

In parallel, tezepelumab targets thymic stromal lymphopoietin (TSLP), an upstream epithelial alarmin involved in type 2 inflammation. By acting upstream of IL-5, IL-4/IL-13, and IgE-mediated pathways, tezepelumab may be particularly attractive in patients with persistent asthma or sinonasal disease despite anti–IL-5/IL-5R therapy. Preliminary reports and small retrospective experiences suggest possible improvement in asthma control, ENT manifestations, lung function, and glucocorticoid tapering after suboptimal response to anti–IL-5/IL-5R agents (75, 76). This rationale is now being tested in non-severe EGPA in the phase 2b RACEMATE trial (NCT06230354).

Finally, combination of monoclonal antibodies targeting type 2 inflammation may be effective in patients with uncontrolled conditions (77, 78). Dupilumab, which blocks IL-4 and IL-13 signaling through IL-4Rα inhibition, may be considered in selected EGPA patients with refractory chronic rhinosinusitis with nasal polyps, uncontrolled airway disease, or persistent type 2 inflammation despite eosinophil-targeted therapy (79, 80). However, it should not be regarded as a vasculitis-directed therapy, as dupilumab may increase circulating eosinophils and EGPA flares have been reported, often preceded by eosinophilia (79). In highly selected refractory cases with complex type 2 disease, combination biologic approaches, such as anti–IL-5/IL-5R therapy plus dupilumab or omalizumab, may theoretically address complementary inflammatory pathways, including eosinophilic, IL-4/IL-13–driven, and IgE-mediated components (77, 78, 81). Nevertheless, evidence remains limited to case reports, small series and retrospective experience; therefore, dupilumab and combination biologics should currently be reserved for expert-center management, with close monitoring of eosinophils and systemic disease activity.

Collectively, these agents reflect an emerging shift toward more tailored and potentially steroid-sparing approaches in EGPA, although further randomized controlled trials are needed to establish their efficacy and optimal positioning.

**FIGURE 2:**
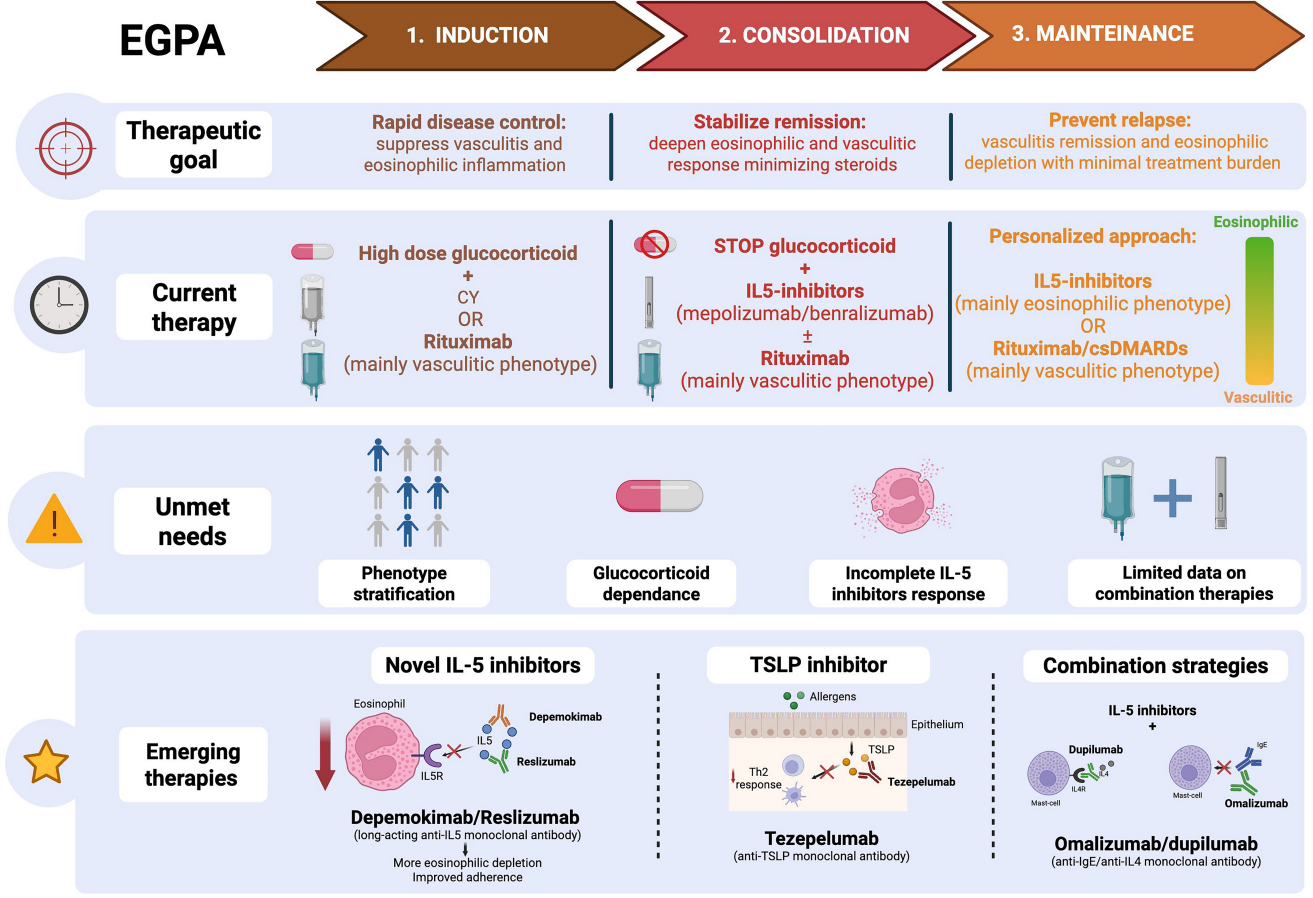
Phase and phenotype-oriented therapeutic framework for eosinophilic granulomatosis with polyangiitis (EGPA).

Figure 2 provides an overview of EGPA management, highlighting the phase-specific therapeutic objectives, current treatment approaches, key unmet needs, and the main emerging eosinophil-targeted strategies.

## 7 Discussion: cross-phase unmet needs and future directions

AAV has evolved from a fatal condition to a chronic, manageable disease, largely due to advances in understanding its immunopathogenesis and the development of targeted therapies. In GPA and MPA, B-cell depletion and complement inhibition have reshaped treatment strategies, while in EGPA, eosinophil-directed therapies have enabled a more phenotype-driven approach.

A phase-oriented approach provides a more clinically relevant framework for understanding and managing AAV, linking pathophysiological mechanisms to specific therapeutic needs (Tables 2, 3).

**TABLE 2:** Unmet needs and potential strategies in GPA and MPA.

Unmet need	Therapeutic strategy	Mechanism of action	Advantages	Limitations
Steroid toxicity	Avacopan	C5aR1 inhibition (complement blockade)	Steroid-sparing; effective disease control; oral administration	Limited long-term data; unclear role in ANCA-negative disease. Evolving hepatotoxicity signal, with markedly higher DILI/VBDS reporting in Japanese real-world/post-marketing data; under evaluation after FDA withdrawal proposal.
Amplification loop (complement)	Vilobelimab	Anti-C5a monoclonal antibody (ligand neutralization)	Potential glucocorticoid-free strategies; broader C5a inhibition	No phase III data; pharmacokinetic limitations
Upstream complement activation	Iptacopan	Factor B inhibition (alternative pathway blockade)	Broad complement inhibition; upstream control of inflammation	Infection risk (encapsulated bacteria); limited clinical data
B-cell persistence	Obinutuzumab	Type II anti-CD20 → enhanced B-cell depletion	More potent and sustained B-cell depletion; improved tissue targeting	Infection risk; hypogammaglobulinemia; uncertain clinical superiority
B-cell reconstitution (BAFF rebound)	Belimumab	Anti-BAFF monoclonal antibody	Prevents autoreactive B-cell re-expansion; improves durability of remission (in combination)	Limited efficacy as monotherapy; delayed onset; additive immunosuppression
Plasma cells	Telitacicept/Daratumumab	BAFF/APRIL blockade/anti-CD38 → plasma cell targeting	Targets autoantibody-producing cells; potential efficacy in refractory disease	Limited evidence; infection risk; hypogammaglobulinemia
Autoantibodies	FcRn inhibitors (e.g., efgartigimod)	Increased IgG degradation via FcRn blockade	Rapid ANCA reduction; non–b-cell depleting; potentially lower immunosuppression	Short duration of effect; limited data in AAV
Immune reset	CAR-T cells	CD19-directed B-cell lineage depletion	Deep immune reprogramming; potential for sustained remission after single treatment	High cost; safety concerns (CRS, infections); limited evidence

**TABLE 3:** Unmet needs and potential strategies in EGPA.

Unmet need	Therapeutic strategy	Mechanism of action	Advantages	Limitations
Glucocorticoid dependence	Mepolizumab/Benralizumab	IL-5/IL-5R blockade → eosinophil depletion	Steroid-sparing; effective in eosinophilic phenotype; reduces relapses	Incomplete response in some patients; limited effect on vasculitic manifestations
Incomplete eosinophilic control	Reslizumab/Depemokimab	Anti–IL-5 (IV weight-based/long-acting)	Higher or prolonged exposure; potential benefit in severe or refractory eosinophilic disease	Limited data in EGPA; similar mechanism to existing therapies
Upstream type 2 inflammation	Tezepelumab	Anti-TSLP → upstream epithelial cytokine blockade	Broader effect beyond eosinophils; effective in heterogeneous type 2 inflammation	Limited evidence in EGPA; role not yet defined
Mixed or heterogeneous phenotype	Dupilumab	IL-4/IL-13 blockade → Th2 pathway inhibition	Effective in airway/ENT disease; targets non–IL-5 pathways	Risk of eosinophilia; possible disease unmasking; limited vasculitic control
Persistent airway/allergic disease	Omalizumab	Anti-IgE → allergic pathway modulation	Effective in allergic asthma phenotype; improves respiratory symptoms	No effect on systemic vasculitis; limited evidence in EGPA
Heterogeneous or refractory disease	Combination biologics (e.g., anti–IL-5 + dupilumab/omalizumab)	Multi-target approach (eosinophils + Th2 pathways)	Addresses complex/mixed phenotypes; potential additive effects	Very limited evidence; safety concerns; high cost
Lack of long-term disease control	Depemokimab	Long-acting anti–IL-5	Reduced dosing frequency; improved adherence	Ongoing trials; long-term efficacy unknown

Future management of AAV will likely move toward a personalized, biomarker-driven model aimed at achieving sustained remission with minimal treatment burden.

Across all phases, three outcomes repeatedly intersect in AAV management: active vasculitis, treatment-related toxicity, and irreversible organ damage. These domains are not sequential but deeply interconnected, and therapeutic success cannot be defined by remission alone. A modern treatment strategy should therefore aim not only for control of inflammation, but for preservation of organ function, reduction of infection burden, minimization of glucocorticoid exposure, and limitation of long-term treatment-related harm.

In conclusion, the phase-oriented framework should not be interpreted as a linear escalation ladder, but as a critical tool for matching therapeutic ambition to evidence strength and patient-level risk. Established strategies such as rituximab, avacopan, and plasma exchange already require individualized trade-offs between relapse prevention, toxicity, feasibility, and cost, whereas emerging approaches such as FcRn inhibition and CAR-T-cell therapy should remain clearly labeled as promising but incompletely validated until controlled and long-term data are available (5, 14, 19, 23, 24, 34, 37, 55, 61–63, 82, 83).
